# Antibodies to a strain-specific citrullinated Epstein-Barr virus peptide diagnoses rheumatoid arthritis

**DOI:** 10.1038/s41598-018-22058-6

**Published:** 2018-02-27

**Authors:** Nicole Hartwig Trier, Bettina Eide Holm, Julie Heiden, Ole Slot, Henning Locht, Hanne Lindegaard, Anders Svendsen, Christoffer Tandrup Nielsen, Søren Jacobsen, Elke Theander, Gunnar Houen

**Affiliations:** 1 Department of Autoimmunology and Biomarkers, Statens Serum Institut, Artillerivej 5, 2300 Copenhagen S, Denmark; 2 Department of Rheumatology, Glostrup Hospital, Nordre Ringvej 57, 2600 Glostrup, Denmark; 3 Department of Rheumatology, Frederiksberg Hospital, Nordre Fasanvej 57, 2000 Frederiksberg, Denmark; 4 Department of Rheumatology, Odense University Hospital, Søndre Boulevard 29, 5000 Odense C, Denmark; 5 Epidemiology, Biostatistics and Bio-demography, Institute of Public Health, University of Southern Denmark, Campusvej 55, 5230 Odense M, Denmark; 6 Copenhagen Lupus and Vasculitis Clinic, Center for Rheumatology and Spine Diseases, Rigshospitalet, Blegdamsvej 9, 2100 Copenhagen, Denmark; 7 Department of Rheumatology, Skåne University Hospital, Lund University, 20502 Malmö, Sweden

## Abstract

Rheumatoid arthritis (RA) is a chronic systemic autoimmune disease. Anti-citrullinated protein antibodies (ACPA) are crucial for the serological diagnosis of RA, where Epstein-Barr virus (EBV) has been suggested to be an environmental agent in triggering the onset of the disease. This study aimed to analyse antibody reactivity to citrullinated EBV nuclear antigen-2 (EBNA-2) peptides from three different EBV strains (B95-8, GD1 and AG876) using streptavidin capture enzyme-linked immunosorbent assay. One peptide, only found in a single strain (AG876), obtained a sensitivity and specificity of 77% and 95%, respectively and showed high sequence similarity to the filaggrin peptide originally used for ACPA detection. Comparison of antibody reactivity to commercial assays found that the citrullinated peptide was as effective in detecting ACPA as highly sensitive and specific commercial assays. The data presented demonstrate that the citrullinated EBNA-2 peptide indeed is recognised specifically by RA sera and that the single peptide is able to compete with assays containing multiple peptides. Furthermore, it could be hypothesized that RA may be caused by (a) specific strain(s) of EBV.

## Introduction

Rheumatoid arthritis (RA) is an autoimmune disease of chronic nature that affects approximately 1% of the world’s population. RA is characterised by inflammation of the synovial joints^1^. Being a serologic marker for the diagnosis of RA, anti-citrullinated protein antibodies (ACPA) are characteristic for RA, which recognise citrullinated proteins in the joints among others^2^. Moreover, ACPAs are associated with a progressive disease course^3^.

ACPAs are specific for epitopes containing the amino acid citrulline (Cit), which is the result of a post-translational modification catalysed by peptidyl arginine deiminases^4^. ACPA has been found to recognise a number of different citrullinated substrates^5–12^. The fact that several non-homologous citrullinated proteins have been recognised by ACPA confirms that these antibodies are cross-reactive^6,10,13,14^.

ACPAs have been detected in 60–80% of RA sera, dependent on the assay used for detection and the cohort analysed^15–19^. Several generations of ACPA assays exist, all attempting to increase assay sensitivity and specificity^8,15,16,19–21^. The substrates used in the assays are unknown for proprietary reasons. Recently, a cyclic citrullinated peptide (CCP) 3.1 assay was introduced. What separates the CCP 3.1 assay from the remaining assays, is that it detects IgA and IgG ACPAs, whereas the remaining assays only detect IgG. Nevertheless, IgA ACPA levels are in general of low titer compared to IgG levels^10,15,22,23^. Moreover, studies indicate that detection of both classes is not useful for diagnostic purposes, since IgA ACPAs rarely are detected without the presence of IgG ACPA^15,22,23^. Hence, the second generation of CCP assays is still regarded as the golden standard for ACPA detection.

The application of viral peptides for detection of ACPA has been described as well^10,24–26^. Studies by Pratesi and coworkers describe the reactivity of ACPA to multiple citrullinated peptides originating from EBNA-1^25^. Using repetitive Arg-Gly-containing peptides as substrates, a peptide corresponding to amino acids 35–58 from EBNA-1, obtained a sensitivity of 45%, when replacing Arg with Cit^24^. Similar studies have been conducted using a repetitive Arg-Gly peptide from EBNA-2, corresponding to amino acids 338–358, where Arg was replaced with Cit^26^. Using this peptide, a sensitivity of 66% was obtained. Furthermore, the antibodies to the EBNA-2 peptide were found to associate with erosive arthritis. Based on these findings, it was hypothesised that viral proteins may be involved in the generation of the ACPA response^25,26^. We recently used a systematic screening approach to analyse ACPA reactivity to substituted Cit-Gly-containing peptides covering the complete EBNA-1 protein^10^. The peptide ARGGSRERARGRGRG-Cit-GEKR, obtained a relatively high sensitivity of 53%, whereas a peptide panel, containing the five most reactive peptides yielded a sensitivity of 67%^10^.

In this study, we analysed ACPA reactivity to several viral EBNA-2 peptides. Thorough analysis revealed that especially the citrullinated peptide corresponding to amino acids 313–333 of the type 2 strain EBV AG876 was significantly recognised by RA sera. By studying antibody reactivity to the selected peptide, we show that antibodies to the peptide are highly specific and sensitive for RA, making the selected peptide an outstanding substrate for ACPA detection.

## Results

### Selection of Epstein-Barr nuclear antigen proteins for screening

Studies of citrullinated epitopes find that Cit flanked by small neutral amino acids are favored for ACPA detection^6,8,10,27^. Thus, Arg-Gly-containing sequences were selected as point of origin for peptide selection. 35 Arg-Gly motifs were found in EBNA-2, EBNA-3A, EBNA-3B, EBNA-3C and EBNA-LP of the three EBV strains (Table 1). As seen, EBNA-LP contained two Arg-Gly motifs, whereas EBNA-2 contained 14 non-homologous Arg-Gly motifs among the three strains. Moreover, high sequence similarity was found between the EBV GD1 and the EBV B95-8 strains due to a high homology between the two (92% sequence similarity). Based on the current findings, the EBNA-2 protein, containing the highest number of different Arg-Gly motifs, was selected for further analysis.

**Table 1:** Arg-Gly motifs located in EBNA proteins in three Epstein-Barr virus strains.

Protein	Sequence	Strain	Strain match	Sequence match*
EBNA-2	MANYIVRQSRGDRGLILPQG	B95-8, GD1	2/3	100%
	YIVRQSRGDRGLILPQGPQT	B95-8, GD1	2/3	100%
	QPSKTQGQSRGQSRGRGRGR	B95-8, GD1	2/3	100%
	TQGQSRGQSRGRGRGRGRGR	B95-8, GD1	2/3	100%
	GQSRGQSRGRGRGRGRGRGK	B95-8, GD1	2/3	100%
	SRGQSRGRGRGRGRGRGKGK(B95-8) SRGQSRGRGRGRGRGRGKSR(GD1)	B95-8, GD1	2/3	90%
	GQSRGRGRGRGRGRGKGKSR(B95-8) GQSRGRGRGRGRGRGKSRDK(GD1)	B95-8, GD1	2/3	80%
	SRGRGRGRGRGRGKGKSRDK(B95-8) SRGRGRGRGRGRGKSRDKQR(GD1)	B95-8, GD1	2/3	70%
	GRGRGRGRGRGKGKSRDKQR(B95-8) GRGRGRGRGRGKSRDKQRKP(GD1)	B95-8, GD1	2/3	60%
	MAQYLLRNARGQQGLLRPLG	AG876	1/3	—
	KQGPDQGQGRGRWRGRGRSK	AG876	1/3	—
	DQGQGRGRWRGRGRSKGRGR	AG876	1/3	—
	GQGRGRWRGRGRSKGRGRMH	AG876	1/3	—
	WRGRGRSKGRGRMHKLPEPR	AG876	1/3	—
EBNA-LP	RRHRSPSPTRGGQEPRRVRR	B95-8, GD1, AG876	3/3	100%
	VVSGSPSGPRGDRSEGPGPT	B95-8, GD1, AG876	3/3	100%
EBNA-3A	EDAHLEPSQRGKKRKRVDDD	AG876	1/3	—
	AQAWNAGFLRGRAYGIDLLR(B95-8) AQAWNAGFLRGRAYGLDLLR(GD1) AQAWNAGLLRGRAYGQDLLR(AG876)	B95-8, GD1, AG876	2/3 1/3	GD1: 95% AG876: 90% —
	DQLPGVPKGRGACAPVPALA	AG876	1/3	—
	DEDLPCIVSRGGPKVKRPPI	B95-8, GD1	2/3	100%
	QGKEVLEKARGSTYGTPRPP	B95-8, GD1	2/3	100%
	GMAYPLHEQRGMAPCPVAQA	GD1	1/3	—
EBNA-3B	NEEIDLAYARGQAMNIEAPR SEETDLAYARGLAMSIEAAR(AG876)	B95-8, GD1, AG876	3/3	GD1: 100% AG876: 75%
	VPPVPRQRPRGAPTPTPPPQ VPPVPRQRPRGAPTPTPPPQ(B95-8) VPPVPRQRSRGAPTPTPPPQ(GD1) GPPTAMQRPRGAPTPMPPPQ(AG876)	B95-8, GD1 B95-8, GD1, AG876	2/3 3/3	100% GD1: 95% AG876: 75%
	VPQQPRAGRRGPCVFTGDLG(B95-8) VPQQPQAGRRGPCVYTGDLG(GD1)	B95-8, GD1	2/3	90%
	APTEYTRERRGVGPMPPTDI(B95-8) EPTEYTRERRGVGPMPPTDI(GD1)	B95-8, GD1	2/3	95%
EBNA-3C	DSRQSPDNERGDNVQTTGEH(B95-8) DSIQSPDNARGDDVQNTGEH(AG876)	B95-8, GD1, AG876	3/3	GD1: 100% AG876: 80%
	RDQQPWGQSRGDENRGWMQR(B95-8) RDQQSRGQRRGDENRGWMQR(AG876)	B95-8, GD1, AG876	3/3	GD1: 100% AG876: 85%
	WGQSRGDENRGWMQRIRRRR(B95-8) RGQRRGDENRGWMQRIRRRR(AG876)	B95-8, GD1, AG876	3/3	GD1: 100% AG876: 90%
	ARQRLQDIRRGPLVVEGGVG(GD1) ARQRLQDIRRGPLVAEGGVG(B95-8)	GD1, B95-8, AG876	3/3	B95-8:95% AG876:100%
	AREAEVRFLRGKWQRRYRRI(B95-8) AREAEVRFLRGKWQRRFRRI(AG876)	B95-8, GD1, AG876	3/3	GD1: 100% AG876: 95%
	PNENPYHARRGIKEHVIQNA(B95-8) PNENPYHARRGIKEQVIQKA(AG876)	B95-8, GD1, AG876	3/3	GD1: 100% AG876: 95%
	SMLATGGEPRGDATSETSSD(B95-8) SMLATVGEPRGDATSETSSD(GD1) SMLATGGGPRGDATSETSSD(AG876)	B95-8, GD1, AG876	3/3	GD1: 95% A876: 95%
	PTPPPSRRRRGACVVYDDDV(B95-8) PPPPPSRRRRGACVVYDDDI(AG876)	B95-8, GD1, AG876	3/3	GD1: 100% AG876: 90%
	EPDSRDQQSRGQRRGDENRG	AG876	1/3	—

*Sequence match is calculated relative to the listed EBV strain for the specific peptide.

### Screening of Cit-Gly-containing Epstein-Barr nuclear antigen-2 peptides

To determine ACPA-specific reactivity to the selected Cit-Gly peptides, 21-mer peptides were synthesized and screened for antibody reactivity by streptavidin capture enzyme-linked immunosorbent assay (ELISA). In total, 15 RA and 10 healthy donor (HD) sera were screened for reactivity.

**Figure 1:**
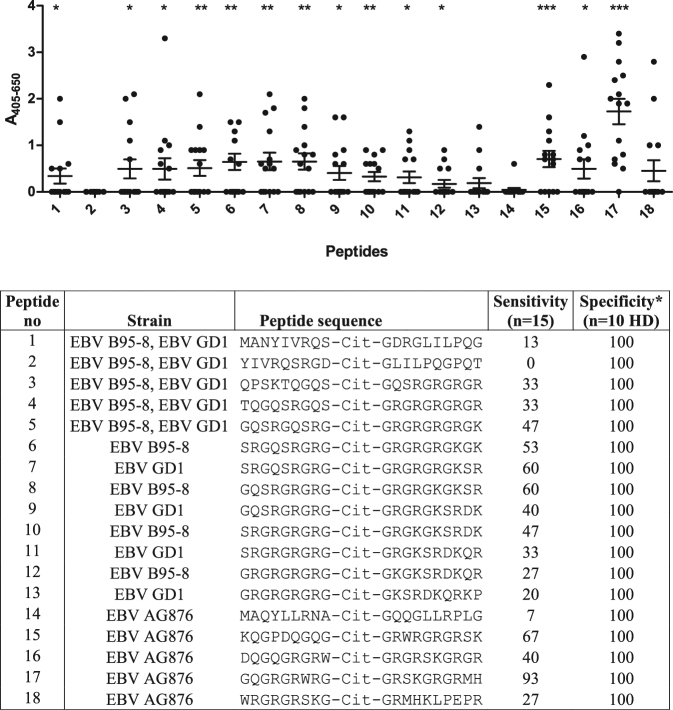
Reactivity of rheumatoid arthritis and healthy donor sera to selected linear EBNA-2 peptides originating from three Epstein-Barr virus strains anslysed by streptavidin capture ELISA. *Specificity is calculated based on the reactivity of 10 healthy donor sera (HD) to the specific peptides. Statistical calculations are performed using the Student’s t-test, where antibody reactivity to healthy controls is used for comparison. a. Reactivity of rheumatoid arthritis sera (n = 15). b. Reactivity of healthy donor sera (n = 10).

As presented in Fig. 1a, significant antibody reactivity was found to the majority of the peptides (1, 3, 4, 5, 6, 7, 8, 9, 10, 11, 12, 15, 16, 17) compared to the HD sera (Fig. 1b). Highly significant reactivity was found to peptide 17 (P < 0.0001), which yielded a sensitivity of 93%. Interestingly, the two peptides, which were not recognised by the RA sera, contained no charged amino acids C-terminal to Cit, which previously has been reported to be essential for ACPA reactivity^13,28,29^. None of the HD sera reacted with the citrullinated peptides, which is illustrated by specificities of 100%.

Peptide 17, yielding the highest sensitivity, was only present in the EBV AG876 strain, whereas peptides 7 (P = 0.0020) and 8 (P = 0.0010) from the EBV GD1 and EBV B95-8 strains, respectively, yielded sensitivities of 60%. As seen, all three peptides contain repetitive Arg-Gly motifs, which previously have been reported for ACPA epitopes^8,10,27,28,30^.

**Figure 2:**
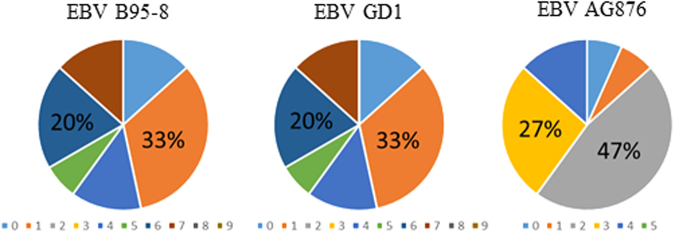
Overlapping antibody reactivities to Cit-Gly-containing peptides among the three Epstein-Barr virus strains; B95-8, GD1 and AG876. The colored fields represent the number of peptides recognised for the individual sera, and hence the degree of overlapping reactivities in a serum pool.

Analyzing the intra-viral cross-reactivity showed that no specific EBV strain was significantly more antigenic than the remaining strains (Fig. 2). Thus, 87% of RA sera recognised peptides from all strains, whereas a single serum only recognised peptides from the EBV AG876 strain and a single serum did not react with any of the peptides. These findings conform to that the presence of Cit-Gly in combination with a favorable structure rather than a specific epitope is essential for antibody reactivity^6,28,30^. Moreover, approximately 33% of the RA sera recognizing peptides from the EBV GD1 and EBV B95-8 strains reacted with a single peptide, whereas more than 50% of the sera recognised four to seven peptides (out of nine peptides in total) (Fig. 2). Similarly, approximately 13% of the RA sera recognised a single peptide from the EBV AG876 strain, whereas more than 70% recognised two to four peptides (out of five peptides in total). These findings confirm that ACPAs are a collection of overlapping and non-overlapping reactivities^14^.

### Screening of modified Epstein-Barr nuclear antigen-2 peptides for optimal antigen presentation

Recently published studies describe that peptide structure and the location of biotin labeling influence ACPA reactivity^10,28,31^. To determine whether the same effect was found for EBNA-2 peptides, linear and cyclic EBNA-2 versions of peptide 17 with a N- and C-terminal biotin labeling were analysed for ACPA reactivity by streptavidin capture ELISA. For preliminary screenings, 10 RA sera and 10 HD sera were screened for ACPA reactivity. Cysteines used for cyclization of the EBNA-2 peptide were introduced in positions five and 19 (GQGR**C**GRWRG-Cit-GRSKGRG**C**RMH-B), which is similar to the Cys residues introduced in the original CCP1 peptide used for detection of ACPA by Schellekens and coworkers^20^.

**Figure 3:**
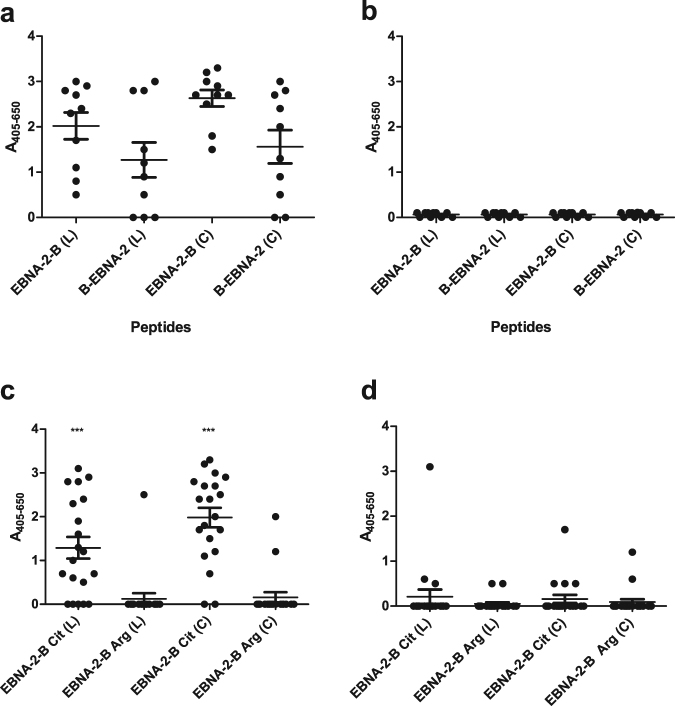
Reactivity of rheumatoid arthritis sera and healthy donor sera to linear and cyclic EBNA-2 peptides and control peptides analysed by streptavidin capture ELISA. (**a**) Reactivity of rheumatoid arthritis sera (n = 10) to cyclic and linear N/C-terminally biotinylated EBNA-2 peptides (amino acids 313–333 of Epstein-Barr virus strain AG876). “B” represents the location of the biotin labeling in relation the “EBNA” peptide. L = linear, C = cyclic. Linear peptide: GQGRGRWRG-Cit-GRSKGRGRMH-B, cyclic peptide: GQGRCGRWRG-Cit-GRSKGRGCRMH-B. Statistical calculations were performed using the Student’s t-test, where antibody reactivity to healthy controls was used for comparison. (**b**) Reactivity of healthy donor sera (n = 10) to linear N/C-terminally biotinylated EBNA-2 peptides. (**c**) Reactivity of rheumatoid arthritis sera (n = 20) to linear and cyclic EBNA-2 peptide linked to a C-terminal biotin (amino acids 313–333 of Epstein-Barr virus strain AG876). Non-citrullinated peptides (Arg) were used at controls. Statistical calculations were performed using the Student’s t-test, where antibody reactivity to non-citrullinated peptides is used for comparison. (**d**) Reactivity of healthy donor sera to linear and cyclic EBNA-2 peptides linked to a C-terminal biotin (n = 20).

As presented in Fig. 3a, notable differences in ACPA reactivity were found to the peptides. The cyclic peptides yielded higher absorbance when compared to the linear peptides (although not significant, P > 0.05), whereas both the linear and the cyclic peptides with a C-terminal biotin labeling yielded higher sensitivities compared to the peptides with a N-terminal labeling (although only significant for the cyclic peptides, P = 0.0182). None of the HD sera reacted with the citrullinated peptides (Fig. 3b). These findings confirm that peptide presentation influence optimal antibody detection, as peptides with a C-terminal biotinylation appear to yield a higher sensitivity, but not at the expense of an altered specificity, as none of the HD sera showed any reactivity. Based on these findings, the cyclic and linear peptides with a C-terminal biotinylation were selected for further studies.

To verify that the RA antibody response is specific for the citrullinated peptides (ACPA-specific) rather than the Arg-containing control peptides (EBV-specific), the reactivity of RA sera and HD sera to citrullinated linear and cyclic peptides and the corresponding Arg-containing (non-citrullinated) peptides, presented by a C-terminal biotin, were analysed by streptavidin capture ELISA. 20 RA sera and 20 HD sera were screened for reactivity. As seen (Fig. 3c), significant ACPA reactivity was found to the citrullinated peptides (linear EBNA-2, P = 0.0002, cyclic EBNA-2, P < 0.0001), compared to the Arg-containing peptides. Except from a single HD serum, none of the HD sera showed notable reactivity to the Arg-containing control peptides (Fig. 3d). Thus, the antibody reactivity found to the citrullinated EBNA-2 peptides is the result of specific reactivity to the citrullinated epitope rather than the EBV peptide itself.

Differences in the reactivity of HD sera to the citrullinated EBV peptides in Fig. 3b and d is ascribed to the difference in number of sera screened (10 vs 20).

### Identification of amino acid residues essential for antibody reactivity to the linear Epstein-Barr nuclear antigen-2 peptide

To determine amino acids essential for ACPA reactivity, nine truncated and substituted peptide versions (with a C-terminal biotin labeling) of the linear EBNA-2 peptide were screened for antibody reactivity. (1 - TPPTNTKQGPDQGQGRGRWR-B, 2 - GRSKGRGRMHKLPEPRRPGP-B, 3 - GRWRG(Cit)GRSKGRGRMHKLPE-B, 4 - GPDQGQGRGRWRG(Cit)GRSKG-B, 5 - Ac-QGRGRWRG(Cit)GRSKGRGRMH-B, 6 - GRGRWRG(Cit)GRSKGRGRM-B, 7 - RGRWRG(Cit)GRSKGRGR-B, 8 - GRWRG(Cit)GRSKGRG-B, 9 - GQGAGRWRG(Cit)GRSKGAGAMH-B, Control Cit - GQGRGRWRG(Cit)GRSKGRGRMH-B, Control Arg - GQGRGRWRGRGRSKGRGRMH-B). In total 10 RA sera and 10 HD sera were screened for antibody reactivity.

**Figure 4:**
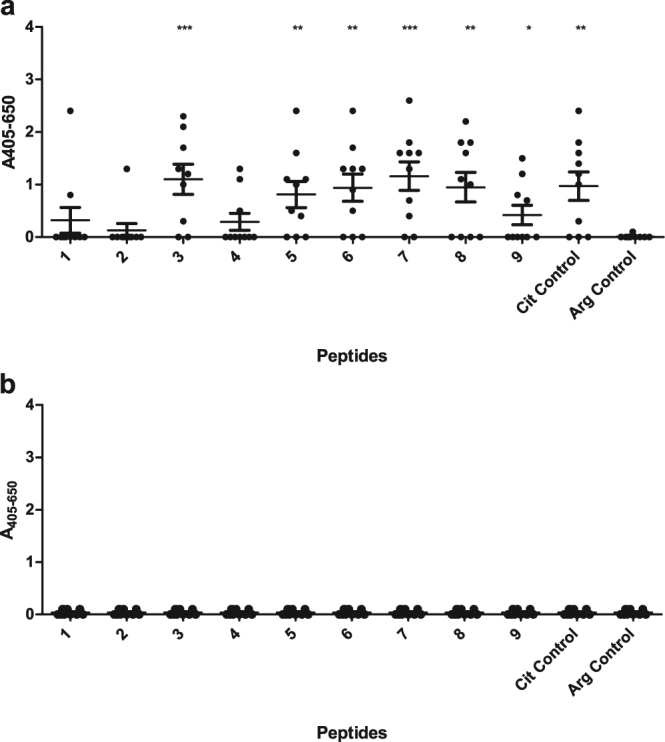
Reactivity of rheumatoid arthritis sera and healthy donor sera to substituted and truncated linear EBNA-2 peptides analysed by streptavidin capture ELISA. Statistical calculations were performed using the Student’s t-test, where antibody reactivity to healthy controls is used for comparison. (**a**) Reactivity of rheumatoid arthritis sera to EBNA-2 peptides (n = 10). (**b**) Reactivity of healthy donor sera to EBNA-2 peptides (n = 10).

As presented in Fig. 4a, the highest sensitivities were obtained when the biotin labeling was located at least five amino acids away from Cit, probably due to steric hindrance. The peptides were significantly recognized by the RA sera compared to the HD sera (except from a single peptide), which conform to previous results obtained. The 15-mer peptide RGRWRG(Cit)GRSKGRGR-B was the shortest peptide (yielding the highest sensitivity) to become significantly recognised by the RA sera (P = 0.0005) compared to the HD sera. When removing Arg in the terminal ends of the peptide, generating the peptide GRWRG(Cit)GRSKGRG, antibody sensitivity was reduced, confirming that charged amino acids are essential for ACPA reactivity, or alternatively that the peptide becomes too short to fold up in a stable conformation. These findings were confirmed when analyzing antibody reactivity to the substituted peptide GQG**A**GRWRG(Cit)GRSKG**A**G**A**MHK, where three Arg residues were replaced with Ala. Even though the Arg residues are not in close proximity to Cit, is the antibody reactivity notably reduced (approximately 60% compared to the control peptide). However, apparently the Arg residues C-terminal to Cit appear to be most important for ACPA reactivity, as the peptide GRWRG(Cit)GRSKGRGRMHKLPE lacks the Arg residue in the N-terminal end, but still yields high antibody sensitivities. Moreover, notable antibody reactivity to this peptide confirm that especially the residues C-terminal to Cit are essential for antibody reactivity. These findings are in accordance to previous results illustrating that especially the C-terminal end of citrullinated peptides are essential for ACPA reactivity^10,28^.

No significant reactivity was determined to the Arg-containing control peptide, (GQGRGRWRGRGRSKGRGRMH), or to the two peptides in close proximity to the citrullinated epitope (TPPTNTKQGPDQGQGRGRWR and GRSKGRGRMHKLPEPRRPGP, underscored amino acids represent amino acids found in the original peptide examined), confirming that the antibody reactivity is a matter of specific ACPA reactivity rather than EBV-specific reactivity. Moreover, none of the HD sera showed notable reactivity to the citrullinated peptides (Fig. 4b). Antibody reactivity to N-terminally biotinylated peptides was analysed as well (see supplementary). In general, no notable difference in reactivity pattern was found to the N-terminally biotinylated peptides compared to the C-terminally biotinylated peptides, although antibody reactivities were significantly reduced, confirming the importance of optimal peptide presentation, when the biotin label is located C-terminally for the EBNA-2 peptide.

### Comparison of the diagnostic potential of the EBNA-2 peptide

To determine the role of the selected linear EBNA-2 peptide (no 17) for ACPA detection, we analysed the antibody reactivity of RA sera and HD sera to the citrullinated peptide with a C-terminal labeling using streptavidin capture ELISA and compared the reactivity of the peptide to two currently available assays; the CCPlus from EuroDiagnostica (CCP2 generation) and CCP3.1 from Inova Diagnostics. In total, 266 sera were screened for reactivity.

**Figure 5:**
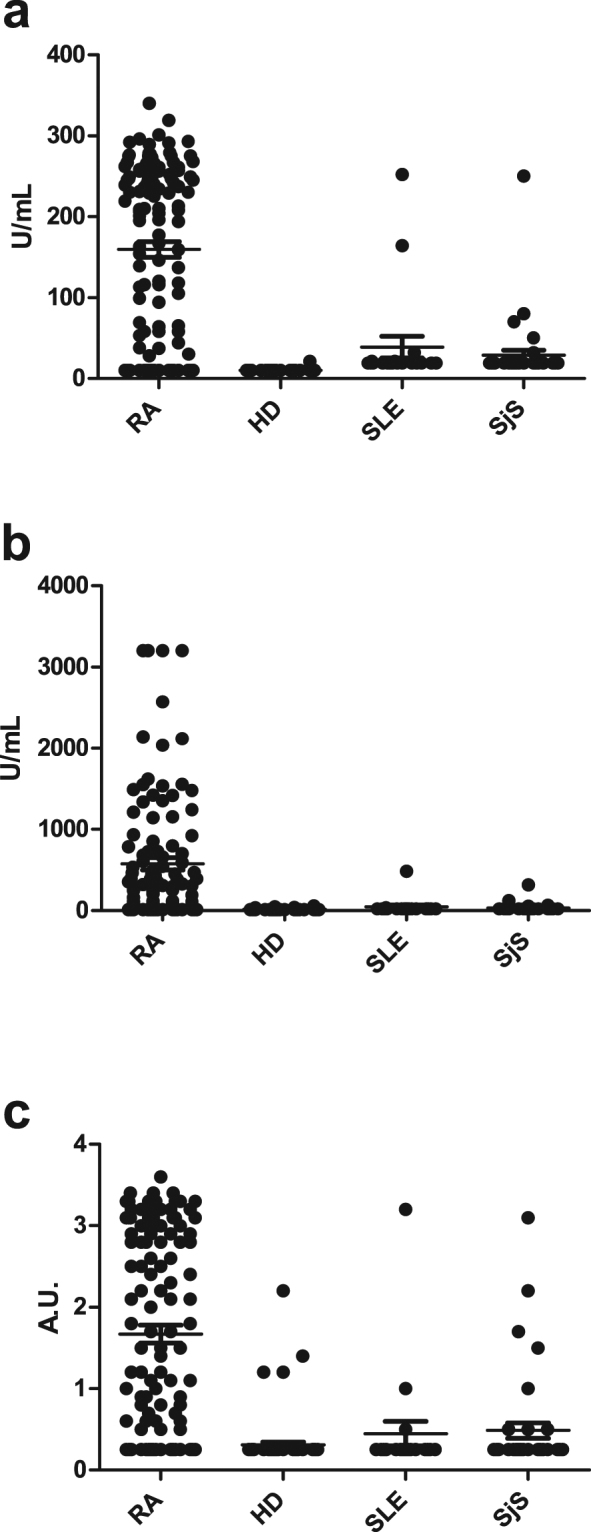
Reactivity of rheumatoid arthritis sera and control sera to the linear EBNA-2 peptide analysed by streptavidin capture ELISA and in the commercial CCPlus and CCP3.1 assays. The following sera were selected for analysis; RA (n = 126), HD (n = 80), SLE (n = 20) and SjS (n = 40). (**a**) Reactivity of RA sera and control sera in the CCP3.1 assay. (**b**) Reactivity of RA sera and control sera in the CCPlus assay. (**c**) Reactivity of RA sera and control sera to the linear EBNA-2 peptide.

Figure 5 represents the reactivity of all sera tested in the three analyses. 97, 90 and 96 sera out of 126 tested positive for reactivity to peptide 17 and in the CCPlus and CCP3.1 assays, respectively, corresponding to sensitivities of 77%, 71% and 76%. Intra-assay concordances found that 64% of RA sera tested positive for reactivity in all assays, whereas 13% were negative for reactivity in all assays. 10% were tested positive for reactivity in two assays, whereas 13% tested positive for reactivity in a single analysis. Out of 16 sera, which were positive for reactivity in a single analysis, 12 were positive for reactivity to peptide 17. To verify that the EBNA-2 peptide is more sensitive than the CCPlus assay, 57 CCPlus-negative sera were analysed for reactivity to the EBNA-2 peptide by streptavidin capture ELISA. Approximately 18% of these sera recognised the EBNA-2 peptide, confirming previous findings (results not shown).

Following sensitivity determination, the specificities of the individual assays were determined (Table 2). 8 HD, 6 SLE and 2 SjS sera tested positive in the CCPlus assay. In contrast, 5 SjS sera and 5 SLE sera and 1 HD serum tested positive in the CCP3.1 assay. 10 SLE sera, 3 SjS sera and 4 HD sera tested positive in the EBNA-2 peptide assay. 5 of the SLE sera tested positive for reactivity to the EBNA-2 peptide and yielded values equal to the cut-off, thus these were regarded as equivocal. Collectively, specificities for the three analyses were within acceptable ranges, as CCP antibodies occasionally are detected in individuals with SLE and SjS^32^. In relation to the HD sera, specificities of 90, 95 and 99% were obtained for the CCPlus assay, the EBNA-2 peptide and the CCP3.1 assay. As seen in Fig. 5, the specificity of the commercial assays appears to be higher when compared to the specificity of the EBNA-2 peptide, this is due to that the scales of the commercial assays are higher, compared to the ELISA assay for the EBNA-2 peptide, which is given in absorbances.

**Table 2:** Antibody reactivity analysed in three different anti-citrullinated protein antibody assays. Sensitivity and specificity are given in %.

	SLE (n = 20)	SLE specificity	SjS (n = 40)	SjS specificity	HD (n = 80)	HD specificity
CCPlus	2	90	6	85	8	90
CCP3.1	5	75	5	88	1	99
EBNA-2	3	85	10 (5)	75 (88)	4	95

SLE; systemic lupus erythematosus, SjS; Sjögren’s syndrome, HD: healthy donor.

### Diagnostic accuracy of the applied assays

Finally, to determine the diagnostic accuracy of each assay, area under the curve (AUC) values for each the EBNA-2 peptide 17, the CCPlus and the CCP3.1 assay were determined (Table 3). The accuracy depends on how well the specific assay separates sera from individuals with RA from HD sera, SLE and SjS.

**Table 3:** Analysis of the diagnostic accuracy of the specific analysis relative to healthy control group and disease controls.

Assay		Area under the curve	Std. Error	95% confidence interval	P-value
*EBNA-2 peptide*					
	RA vs HD	0.8506	0.02666	0.7984–0.9029	<0.0001
	RA vs SLE	0.8242	0.04198	0.7419–0.9065	<0.0001
	RA vs SjS	0.7985	0.03524	0.7294–0.8676	<0.0001
*CCPlus*					
	RA vs HD	0.8369	0.02785	0.7823–0.8915	<0.0001
	RA vs SLE	0.8115	0.04157	0.7300–0.8930	<0.0001
	RA vs SjS	0.8204	0.03196	0.7578–0.8831	<0.0001
*CCP3.1*					
	RA vs HD	0.8810	0.02398	0.8339–0.9280	<0.0001
	RA vs SLE	0.8143	0.04406	0.7279–0.9007	<0.0001
	RA vs SjS	0.8591	0.02759	0.8050–0.9132	<0.0001

A rough guide for classifying the accuracy of a diagnostic test is the traditional academic point system: 0.90–1.0 = excellent, 0.80–0.90 = good, 0.70–0.80 = fair, 0.60–0.70 = poor, 0.50–0.60 = fail. RA, rheumatoid arthritis; SLE, systemic lupus erythematosus; SjS, Sjögren’s syndrome.

For all three assays the AUC values ranged from 0.80–0.88, indicating that all assays are effective in differentiating between RA and controls. In general, a high concordance between the three assays was found, although the AUC for CCP3.1 for HD sera compared to RA sera was slightly higher compared to the CCPlus assay and the EBNA-2 peptide, which conforms to that only a single HD serum reacted in the CCP3.1 assay. In general, the three assays were equally effective in differentiating RA sera from disease controls, as AUC for SLE ranged from 0.81–0.82 and AUC values for SjS ranged from 0.80–0.86.

Collectively, the single EBNA-2 peptide obtained sensitivities, specificities and AUC values similar to the commercial assays, and we conclude that the EBNA-2 peptide indeed is a good substrate for ACPA detection and that a single peptide is able to compete with assays containing numerous citrullinated peptides.

## Discussion

The present study describes the reactivity of RA sera to citrullinated peptides originating from EBV. Based on the presence of Arg-Gly sequences and sequence homology among the three strains analysed, EBNA-2 was selected for further analyses. Peptide 17, which obtained the highest sensitivity, was only found in the EBV AG876 strain. Moreover, results presented in Figs 1 and 3, illustrating RA and HD reactivity to citrullinated and Arg-control peptides, respectively, indicate that the reactivity to the selected peptides are Cit-specific rather than Arg-specific.

Peptide 17 obtained a higher sensitivity compared to the CCPlus assay and a sensitivity similar to the CCP3.1 assay. Both assays contain several peptides, which are unknown due to proprietary reasons. Sensitivities of 71%, 76% and 77% were obtained for the CCPlus assay, CCP3.1 assay and the EBNA-2 peptide, respectively.

Assay specificities illustrate that the EBNA-2 peptide is able to compete with the commercial assays, as high specificities are obtained for all three analyses. When analyzing the reactivity of the SjS sera to the EBNA-2 peptide it was observed that the EBNA-2 peptide reacted with 10 out of 40 sera. Analysing each individual response showed that five of these sera yielded absorbances in the greyzone. By increasing the cut-off for the assay it may be possible to increase the specificity of the assay although at the expense of assay sensitivity.

Several attempts have been initiated to generate a single peptide-based assay for ACPA detection, although without any success. Nevertheless, the linear EBNA-2 peptide was just as efficient in detecting ACPA as the commercial assays, hence the EBNA-2 peptide poses as a strong candidate for future assays detecting ACPA. Previous attempts using other EBNA-1- and EBNA-2-derived peptides, yielded sensitivities up to 60%^10,25,26^, thus the sensitivities are too low to compete with the existing assays.

Several advantages prevail when using a single peptide for antibody detection over assays employing numerous peptides. The use of a single peptide simplifies the assay and ensures more controlled conditions. E.g. addition of several peptides may block binding sites and induce peptide interactions. Moreover, application of single peptides allows for more cost-effective analyses, as only a single peptide needs to be synthesised and coated in ELISA wells.

As previously mentioned, the second generation of ACPA assays, e.g. the CCPlus assay, is regarded as the golden standard for ACPA detection. Several studies have analysed the sensitivity of second and third generation ACPA assays with contradicting results^15,33,34^. Nevertheless, in the current study, the CCPlus assay obtained the lowest sensitivity compared to the CCP3.1 assay and the EBNA-2 peptide. Moreover, the CCP3.1 assay, which detects both IgA and IgG ACPA, apparently should be more sensitive^35^. We did in fact observe that approximately 25% of the sera that were CCPlus-negative were positive in the CCP3.1 assay, nevertheless, these sera primarily tested positive for reactivity to the EBNA-2 peptide as well. Moreover, it has been described that sera negative for IgG ACPA reactivity are IgA ACPA-negative as well, hence it has been concluded that the reactivity in the CCP3.1 assay is ascribed to IgG reactivity. Thus, our findings illustrate that the CCP3.1 assay does not appear to be more sensitive than the EBNA-2 peptide.

Currently, the serology domain of ACR criteria comprises testing of ACPA and RF. However, the detection of ACPA has largely replaced the RF as the most helpful biomarker in the diagnosis of RA. Given the characteristics of ACPA, which is very specific for RA, studies find that ACPA has higher specificity than RF for RA. Collectively, ACPA has some benefits as compared to RF, although these serological biomarkers do not detect the same compounds. However, many prefer to use both markers, which ultimately may increase the overall sensitivity for RA. Nevertheless, as seen using the EBNA-2 peptide, only a small percentage (9 out of 126 RA sera in total), which tested negative for antibody reactivity, were RF-positive, indicating that for future detection, the single EBNA-2 peptide is the most reliable assay and appears to outcompete the RF assays, making it possible to use a single serologic assay when confirming the RA diagnosis along with clinical symptoms. Collectively, several studies find that ACPA has a notably higher sensitivity and specificity for RA, good predictive validity, good reproducibility and stability. Hence, given its superior performance characteristics, ACPA is emerging as the most useful single serologic assay for the confirmation of a clinical diagnosis of RA.

Based on the current findings, the role of EBV in the onset of RA once again yields attention. EBV infects most of the world’s population and is associated with the onset of several diseases, but only limited information is available about how the different EBV strains may influence diseases. Interestingly, the genomic variations that appear to separate EBV strains into type 1 (B95-8, GD1 strain) or type 2 (AG876 strain) subtypes, are defined almost exclusively by variation of EBNA-2 and EBNA-3 genes, and occasional geographical variations^36,37^. Results presented in this study indicate that EBV may be involved in the onset of RA and that the detected antibodies are specific for the citrullinated EBNA-2 peptide. Based on the current studies it may be hypothesized that EBV contributes to the onset of RA. Further studies remain to determine whether a linkage is present between the onset of RA and the type of EBV strain a person is infected with.

## Materials and Methods

Synthetic peptides (Schäfer-N, Lyngby, Denmark) were synthesized using traditional Fmoc solid-phase peptide synthesis^38^. Cyclization of selected peptides was performed by traditional air oxidation. Following oxidation, all peptides were purified by reverse-phase high-performance liquid chromatography and peptide identity was confirmed with liquid chromatography-mass spectrometry. The peptides were synthesized based on the Swiss-Prot id: P12978.1, Q3KSV2.1 and Q69022.1, covering the complete EBV protein EBNA-2 from the three strains; EBV B95-8, EBV GD1 and EBV AG876, respectively. Points of origin for generation of the synthetic peptides, were identified based on Arg-Gly motifs in the three EBV strains.

### Patient samples

126 RA sera, which were diagnosed based on the American College of Rheumatology (ACR) classification criteria, were analysed in this study^39,40^. These sera were evaluated with respect to RF and ACPA/anti-CCP2 levels. RA sera were from collaborators at Danish hospitals (Department of Rheumatology, Glostrup Hospital, Department of Rheumatology, Frederiksberg Hospital, Department of Rheumatology, Odense University Hospital and Epidemiology, Biostatistics and Bio-demography, Institute of Public Health, University of Southern Denmark). In addition, 57 RF-negative and CCP-negative RA sera were analysed in this study. These sera were collected at the Department of Rheumatology, Frederiksberg Hospital, Denmark.

In total, 140 control sera were used in this study originating from healthy donors (HD), individuals with systemic lupus erythematosus (SLE) and sjögren’s syndrome (SjS) (HD, n = 80: SLE, n = 20: SjS: n = 40). Sera from individuals with SLE were diagnosed based on the ACR criteria^41^. Sera from individuals with SjS were diagnosed based on the criteria of the American-European Consensus Group^42^. HD sera were from Rigshospitalet, Copenhagen, Denmark and obtained anonymously. Sera from SjS individuals were provided by the Department of Rheumatology, Skåne University Hospital, Malmö, Sweden. Sera from SLE individuals were provided by Copenhagen Lupus and Vasculitis Clinic, Center for Rheumatology and Spine Diseases, Rigshospitalet, Copenhagen, Denmark. The study was approved by the national committee on health research ethics, Copenhagen, Denmark (Project ID:19980024 PMC and H-15009640) and all experiments were performed in accordance with relevant guidelines and regulations. Moreover, informed consent from all patients contributing to this study was obtained.

### Antibody detection using streptavidin capture enzyme-linked immunosorbent assay

Maxisorp microtiter plates (96-wells) (Nunc, Roskilde, Denmark) were incubated with streptavidin (1 µg/mL) (Sigma Aldrich, St Louis, Mo, USA) diluted in carbonate buffer (15 mM Na_2_CO_3_, 35 mM NaHCO_3_, 0.001 % phenolred, pH 9.6) (Statens Serum Institut (SSI) Diagnostica, Hillerød, Denmark) for 2 hours (h) at room temperature (RT), where after the plate was incubated with biotinylated EBV peptides (1 µg/mL) diluted in PBS (10 mM Na_2_HPO_4_/NaH_2_PO_4_, 0.15 M NaCl, pH 7.2) (SSI Diagnostica, Hillerød, Denmark) for 2 h at RT. Incubations with patient sera/antibodies diluted in Tris-Tween-NaCl (TTN) buffer (0.05 M Tris, 0.3 M NaCl, 1 % Tween 20, pH 7.4) (SSI Diagnostica, Hillerød, Denmark) were conducted for 1 h at room temperature, followed by washing of plates with TTN (3x1 minute). Patient sera (1:200) and alkaline phosphatase (AP)-conjugated goat-anti human IgG (Sigma Aldrich, St Louis, Mo, USA) (1 µg/mL) were added to all wells. Antibody levels were measured by adding *p-*nitrophenylphosphate (Sigma Aldrich, St Louis, Mo, USA) (1 mg/mL) diluted in AP substrate buffer (1M diethanolamine, 0.5 mM MgCl_2_, pH 9.8) (SSI diagnostica, Hillerød, Denmark) to the wells. The absorbance was measured at 405–650 nm, using a microtiter plate reader (Molecular Devices, Menlo Park, CA, USA). Each sample was measured in duplicates. To ensured least possible intra-assay variations, a positive control peptide containing a given specific citrullinated peptide, and an ACPA-positive donor pool were included on each plate. Intra-assay variations within a −/+ range of 0.5 absorbance units were accepted.

### Data interpretation

Sample results were corrected for non-specific reactivity using wells without peptide, and the resulting background levels were subtracted before final data analysis. Based on preliminary screenings, analysing the reactivity of HD sera to selected EBV peptides, a non-specific reactivity of 5% (specificity of 95%) was tolerated and selected as cut-off. Based on these screenings, a cut-off of 0.5 AU for each sample analysed was introduced. Thus, readings of 0.5 AU or above were regarded as positive, whereas samples below were regarded as negative. Readings of 0.5 AU were regarded as equivocal, but ultimately positive.

For generation of ROC curves and calculation of AUCs, readings below cutoff were set to a fixed value. E.g. cutoffs of 25 U/mL, 20 U/mL and 0.5 AU were used for the CCPlus assay, CCP3.1 assay and the EBNA-2 peptide, respectively, and values of 24 U/mL, 19 U/mL and 0.4 AU for all samples below cutoff were used for the three assays, respectively.

Statistical calculations were performed using duplicate measurements of RA and control sera. The values obtained in this study were compared further by using the two-tailed Student’s t-test. The following symbols are used to illustrate statistical significance; (*P < 0.05), (**P < 0.01), (***P < 0.001).

## Electronic supplementary material

Supplementary Information
