# Supplementary material for: Antibodies to a strain-specific citrullinated Epstein-Barr virus peptide diagnoses rheumatoid arthritis
Source: Sci Rep. 2018 Feb 27;8:3684. doi: 10.1038/s41598-018-22058-6 (PMC5829227; doi:10.1038/s41598-018-22058-6)
Supplement: Supplementary file 1 — Supplementary Information [file 41598_2018_22058_MOESM1_ESM.pdf]

Antibodies to a strain-specific citrullinated Epstein-Barr virus peptide diagnoses rheumatoid arthritis

Nicole Hartwig Trier*[1], Bettina Eide Holm[1], Julie Heiden[1], Ole Slot[2], Henning Locht[3], Hanne Lindegaard[4], Anders Svendsen[5], Christoffer Tandrup Nielsen[6], Søren Jacobsen[6], Elke Theander[7], Gunnar Houen[1]*

[1] Department of Autoimmunology and Biomarkers, Statens Serum Institut, Artillerivej 5, 2300 Copenhagen S, Denmark

[2] Department of Rheumatology, Glostrup Hospital, Nordre Ringvej 57, DK-2600 Glostrup

[3] Department of Rheumatology, Frederiksberg Hospital, Nordre Fasanvej 57, 2000 Frederiksberg, Denmark

[4] Department of Rheumatology, Odense University Hospital, Søndre Boulevard 29, 5000 Odense C, Denmark

[5] Epidemiology, Biostatistics and Bio-demography, Institute of Public Health, University of Southern Denmark, Campusvej 55, 5230 Odense M, Denmark

[6] Copenhagen Lupus and Vasculitis Clinic, Center for Rheumatology and Spine Diseases, Rigshospitalet, Blegdamsvej 9, 2100 Copenhagen, Denmark

[7] Department of Rheumatology, Skåne University Hospital, Lund University, 20502 Malmö, Sweden

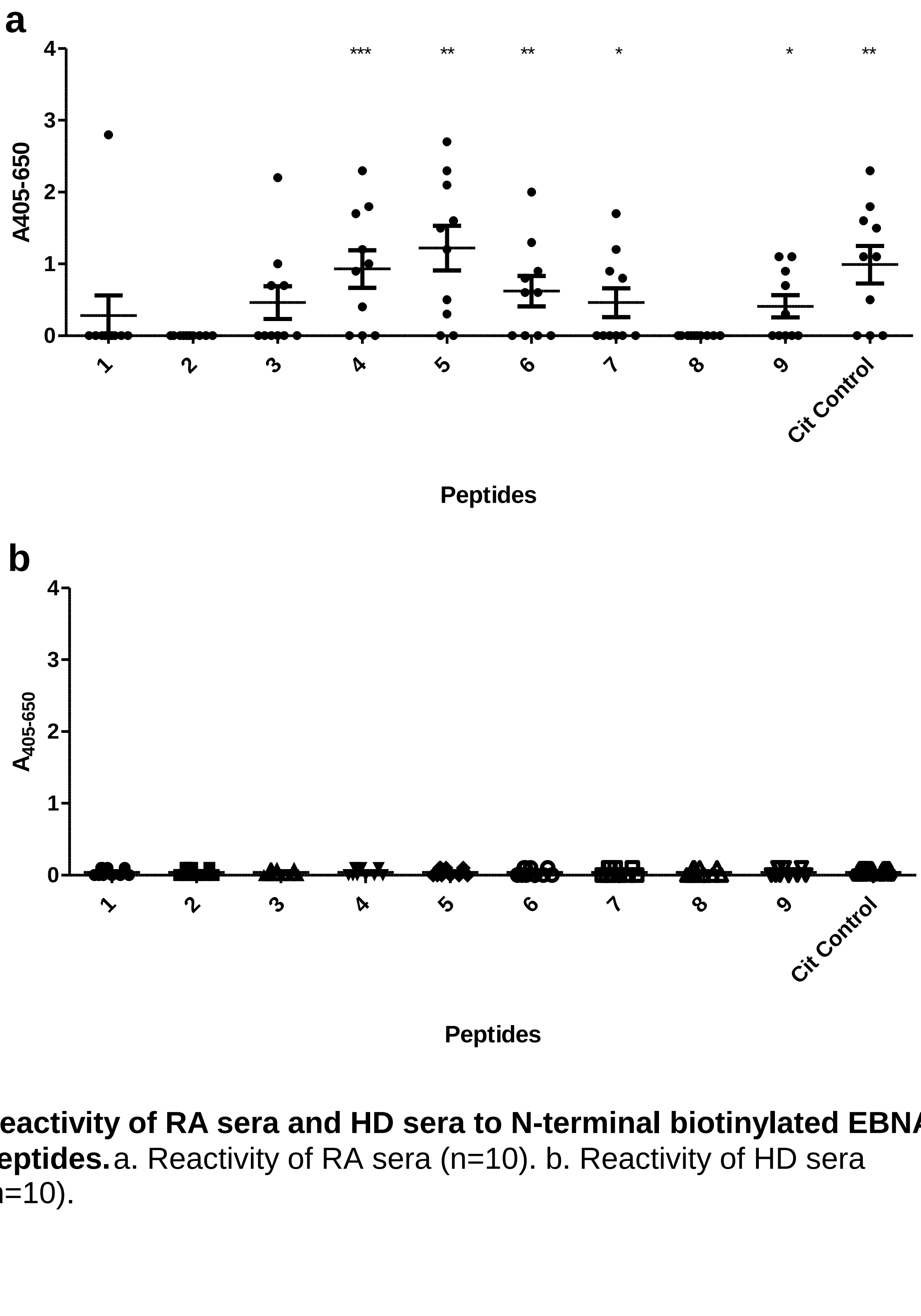

Reactivity of RA sera and HD sera to N-terminal biotinylated EBNA-2 peptides. a. Reactivity of RA sera (n=10). b. Reactivity of HD sera (n=10).
